# Constructing cities, deconstructing scaling laws

**DOI:** 10.1098/rsif.2014.0745

**Published:** 2015-01-06

**Authors:** Elsa Arcaute, Erez Hatna, Peter Ferguson, Hyejin Youn, Anders Johansson, Michael Batty

**Affiliations:** 1 Centre for Advanced Spatial Analysis (CASA), University College London, London, UK; 2 Center for Advanced Modeling, The Johns Hopkins University, Baltimore, MD 21209, USA; 3 The Institute for New Economic Thinking at the Oxford Martin School, University of Oxford, Oxford, UK; 4 Santa Fe Institute, Santa Fe, MN 87501, USA; 5 Department of Civil Engineering, University of Bristol, Bristol, UK

**Keywords:** power-laws, scaling laws, urban indicators, city boundaries

## Abstract

Cities can be characterized and modelled through different urban measures. Consistency within these observables is crucial in order to advance towards a science of cities. Bettencourt *et al*. have proposed that many of these urban measures can be predicted through universal scaling laws. We develop a framework to consistently define cities, using commuting to work and population density thresholds, and construct thousands of realizations of systems of cities with different boundaries for England and Wales. These serve as a laboratory for the scaling analysis of a large set of urban indicators. The analysis shows that population size alone does not provide us enough information to describe or predict the state of a city as previously proposed, indicating that the expected scaling laws are not corroborated. We found that most urban indicators scale linearly with city size, regardless of the definition of the urban boundaries. However, when nonlinear correlations are present, the exponent fluctuates considerably.

## 1. Introduction

Cities are the outcome of intricate social and economic dynamics, shaped by geographical, cultural and political constraints. There is however little understanding on how all the different features interweave and co-evolve. Certain properties such as morphological attributes, e.g. fractality of cities [1,2], Zipf distributions of city sizes [3,4] and population growth laws [5–8], seem to transcend contextual constraints although debate remains with respect to the universality of some of these characteristics [9–11].

**Figure 1.**
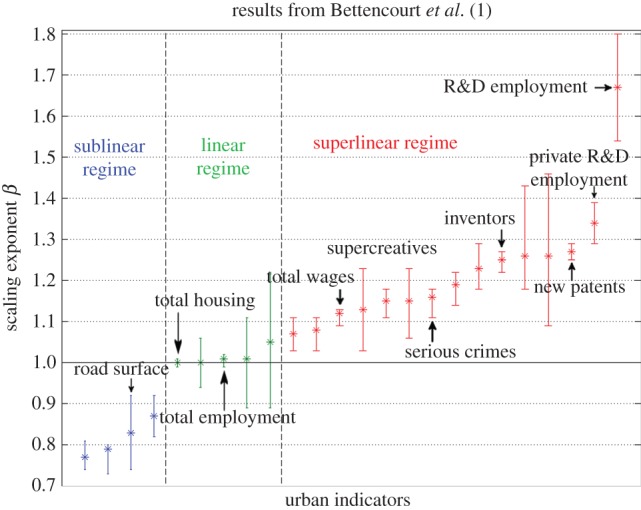
Exponents with 95% CI for different urban indicators found for the USA, Germany and China in reference [14]. These are colour-coded according to their regime. (Online version in colour.)

In the past decade, drawing from an analogy with Kleiber's law [12,13] which stipulates allometric scaling of the metabolic rate with respect to the mass of an animal, it has been proposed that most urban indicators can be determined in terms of the following ubiquitous scaling law [14–17]1.1
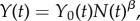
where *Y*(*t*) and *N*(*t*) represent the urban indicator and the population size of a city at time *t* respectively, and *Y*_0_(*t*) is a time-dependent normalization constant. It is conjectured that the nature of the urban observable will unequivocally define one of the three universal categories to which the scaling exponent *β* belongs: (i) *β* < 1, a sublinear regime given by economies of scale associated with infrastructure and services, e.g. road surface area; (ii) *β* ≈ 1, a linear regime associated with individual human needs, e.g. housing and household electrical consumption and (iii) *β* > 1, a superlinear regime associated with outcomes from social interactions, e.g. number of patents and income [18]. Observations in the USA, Germany and China [15] seem to provide empirical evidence supporting the conjectured values for the exponent in equation (1.1). These results together with their confidence intervals (CIs) are pictured in figure 1. These are punctual values for a single predetermined definition of urban areas: metropolitan statistical areas (MSAs) in the USA, and larger urban zones (LUZs) in Europe. These definitions were designed to incorporate urbanized and economic functional areas, but they are not necessarily consistent with one another as no consensus exists on how cities should be defined.

In this article, we investigate the extent to which in England and Wales (E&W)^1^, urban indicators can be estimated on the basis of size alone according to equation (1.1), regardless of constraints, such as intercity interactions, globalization or simply historical dependencies. Instead of limiting the analysis to a single predefined definition of cities such as LUZ, we define a simple procedure that produces a system of cities by aggregating small statistical units. We chose this approach for the following reasons: (i) the LUZ selection of cities is very small as only 21 cities in E&W are considered, whereas important cities such as Oxford and Reading are missing, leading to a small sample space; (ii) the procedure can be easily reproduced in other countries and it thus allows for a consistent comparison with other urban systems, and more importantly, (iii) this methodology provides a set of different realizations of the urbanized space, serving as a laboratory to explore the sensitivity of the urban indicators to a comprehensive set of different city and metropolitan area demarcations in E&W, leading to a more rigorous framework to test urban hypotheses. For the curious reader who is interested in a direct comparison with the LUZ definition, the results of the scaling analysis can be found in the electronic supplementary material, figure S8. The findings for LUZ do not corroborate the expected behaviour reported in reference [14].

There are different methods to reconstruct urban systems, for example through urban growth [8,19–21], or other methods using percolation and diffusion-limited aggregation [22–25]. In this paper, we apply a simple methodology that consists of two steps. The first step uses a clustering algorithm parametrized by population density. This gives rise to settlements defined through urban morphology only. For a particular range of the population density threshold, a good representation of the extent of cities can be recovered. Nevertheless, we do not limit our analysis to this range, so that we are able to analyse the robustness of the scaling exponent to the different configurations of the urban extent.

The second step consists of defining metropolitan areas based on the clusters that were obtained in the first step. This is achieved by adding areas to cities according to a commuting threshold. The approach is similar to the way other definitions of metropolitan areas, such as MSAs, are defined but instead of using a single commuting threshold (such as the typical value of about 30%), we once again define cities over the whole range of commuting thresholds.

We present the results for plausible cases of cities and metropolitan areas as well as for the entire range of density and commuting thresholds.

## 2. Data

Most of the variables used in the analysis come from the 2001 UK census dataset, produced by the Office for National Statistics. The data are given at the level of *wards*, which are the smallest geographical units in the census data across many variables. E&W consists of 8850 wards that reflect the political geography of the country at a fine resolution and have similar populations owing to the need to maintain equality of representation in political elections.

Data on household income were taken from the UK census experimental statistics for 2001/2002, and it corresponds to estimates produced using a model-based process. Infrastructure data, such as the area of roads, paths and buildings, come from the 2001 Generalized Land Use Database. Finally, data on patents were provided by the Intellectual Property Office at the postcode level, for the years 2000 to 2011. Each of the tables from which the indicators were obtained is described in detail in the electronic supplementary material.

## 3. Clustering through density thresholds: cities

**Figure 2.**
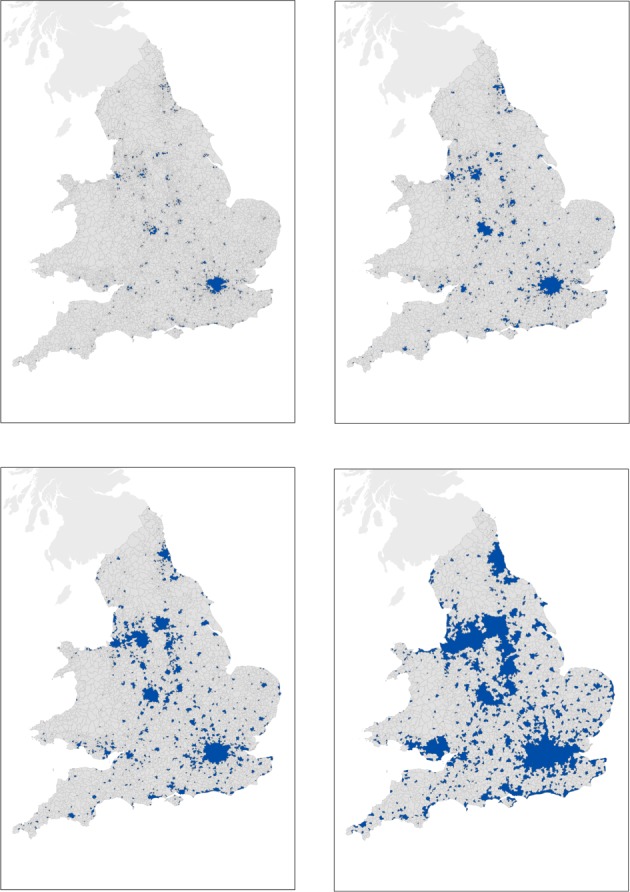
Sample of configurations of cities for four different density cut-offs. From top left to bottom right: *ρ* = 40, *ρ* = 24, *ρ* = 10 and *ρ* = 2 prs ha^−1^. (Online version in colour.)

The algorithm described in this section gives rise to configurations of clusters representing cities and smaller settlements in terms of their morphological extent. We use population density as the main parameter, because this is an intrinsic property of urbanized spaces. The unit of agglomeration for our algorithm is a ward (see the electronic supplementary material for details). We define the parameter for population density *ρ*_0_ to lie within the interval [1;40] persons per hectare. For each integer threshold *ρ*_0_ in the interval, we cluster all adjacent wards with density *ρ*_*w*_ such that *ρ*_*w*_ *_≥_* *ρ*_0_. If a ward *k* has a density *ρ*_*k*_ < *ρ*_0_ but is surrounded by wards such that for each ward *w*, *ρ*_*w*_ ≥ *ρ*_0_, then the ward *k* is also included in the cluster. The resulting city area is hence a continuous surface. We obtain 40 different realizations of systems of cities for E&W, varying from very large clusters containing various settlements, to clusters containing only the core of cities for the highest density values (figure 2).

**Figure 3.**
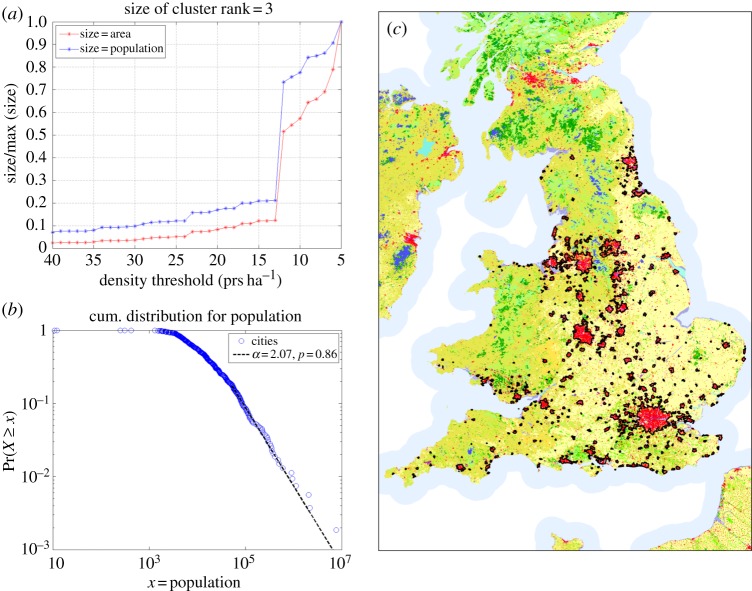
System of cities defined at a density cut-off of *ρ*_c_ = 14 prs ha^−1^. (*a*) Transition of cluster size; (*b*) Zipf distribution of city size; (*c*) the Corine land cover map of E&W: red corresponds to the built area, and the black contours to the clusters defined for *ρ*_c_ = 14 prs ha^−1^.

For a range of densities, the algorithm produces realizations that are in very good agreement with the identified urbanized areas. One of many possible good realizations can be determined by looking at transitions in the cluster sizes resulting from the change in density from high to low values. The largest cluster exhibiting a sharp transition is the third biggest one (rank 3; figure 3*a*), and the jump corresponds to the merging of Liverpool and Manchester. Given that these two cities are very close, we select the density threshold *ρ*_c_ = 14 prs ha^−1^, which is near the transition and before the joining takes place. It is important to note that this choice is not unique, and the properties and results that we will show below hold for a range of choices of *ρ*. The system of cities defined at *ρ*_c_ has a Zipf distribution of city sizes^2^, figure 3*b*, and the boundaries, displayed as black contours in figure 3*c*, show an excellent overlap with the built areas (red clusters in the map) derived from remote sensing [27]. Cities specified at the density of *ρ*_c_ = 14 prs ha^−1^ are therefore a good proxy for a definition of cities *vis à vis* of their morphology, i.e. the urbanized space.

## 4. Clustering through commuting thresholds: metropolitan areas

Metropolitan areas correspond to urban agglomerations linked together through socio-economic functionalities. We construct such areas by considering the density-based cities as destinations of commuter flows. For each city, we add the areas that are the origins of its commuter flows.

In order to include small settlements as origins rather than destinations of commuting flows, we impose a minimum population size on the initial clusters, such that only the larger settlements are considered commuting hubs. The data on commuter flows at the ward level are taken from the 2001 census of the UK [28].

In detail, this second algorithm works as follows. For each density realization *ρ*_0_ ∈ {1, 2, … , 40} prs ha^−1^, we impose a minimum population size cut-off *N* ≥ *N*_0_ for each of the clusters, where *N*_0_ ∈ {0, 10^4^, 5 × 10^4^, 10^5^, 1.5 × 10^5^} individuals.^3^ We remove smaller clusters to allow their constituting wards to be part of larger clusters, as is the case of satellite settlements around London. For every given ward, we compute the percentage of individuals commuting to each of the clusters out of the total number of commuters from the ward. The ward is added to the cluster that receives the largest flow if the flow is above a threshold^4^ *τ*_0_. We investigate all the different realizations for *τ*_0_ ∈ {0, 5, … , 100}% individuals commuting from a ward to a cluster. The extreme value of *τ*_0_ = 100% reproduces the original system without commuting as the percentage of commuters from a given ward cannot exceed 100%. The other extreme value of *τ*_0_ = 0% in which a ward is added to a cluster if a single individual commutes to it, leads to an almost full coverage of E&W, where nearly every ward belongs to a cluster.

**Figure 4.**
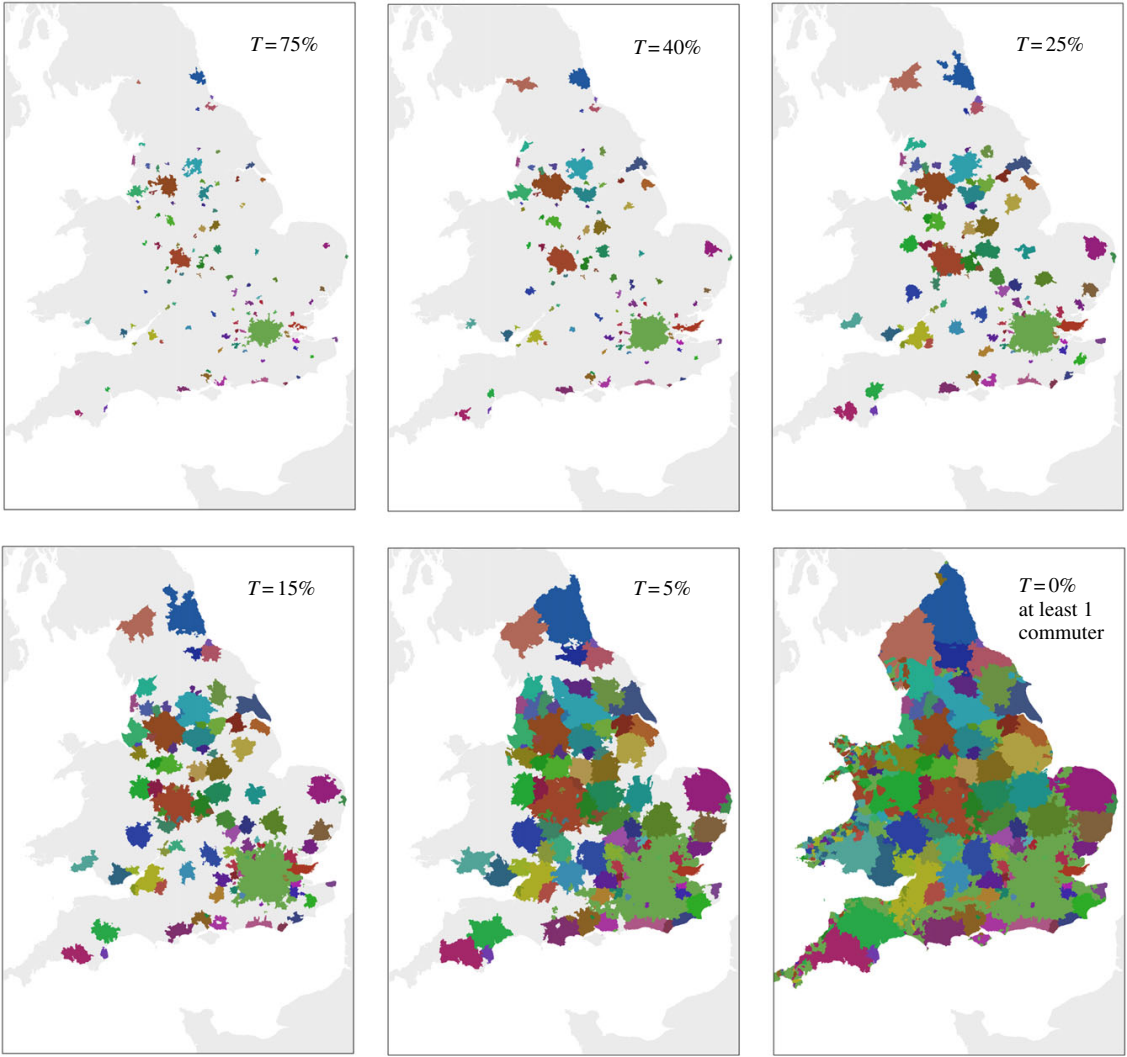
Realizations of metropolitan areas at fixed density cut-off of *ρ*_c_ = 14 prs ha^−1^ and a minimum population size of 5 × 10^4^ individuals for a selection of several commuting flow thresholds *τ*.

Specific realizations for the density cut-off of *ρ*_c_ = 14 prs ha^−1^, a minimum population size of *N* = 5 × 10^4^ individuals and different flow thresholds *τ*, are pictured in figure 4. Notable changes in the configuration of the clusters are observed below the threshold of 50%, indicating that rarely the majority of individuals in a ward will commute to a single cluster. As a result, the realization for a flow of 75% is almost identical to the realization pre-commuting clustering. This method gives rise to more than 2 × 10^4^ realizations of systems of cities that serve as a laboratory to assess the behaviour of the scaling exponent in equation (1.1).

## 5. Results for cities and metropolitan areas

In this section, we focus on the scaling analysis for cities and metropolitan areas in order to make our results comparable to other studies. We already demonstrated that clusters defined at *ρ*_c_ = 14 prs ha^−1^ (figure 3) provide a good proxy for cities, and hence we use this definition in the analysis. Metropolitan areas are commonly understood as cities that include the regions from which at least 30% of the population commute to work. We therefore construct the metropolitan areas through the second clustering method for *ρ*_c_ = 14 prs ha^−1^ and *τ*_0_ = 30%.

**Figure 5.**
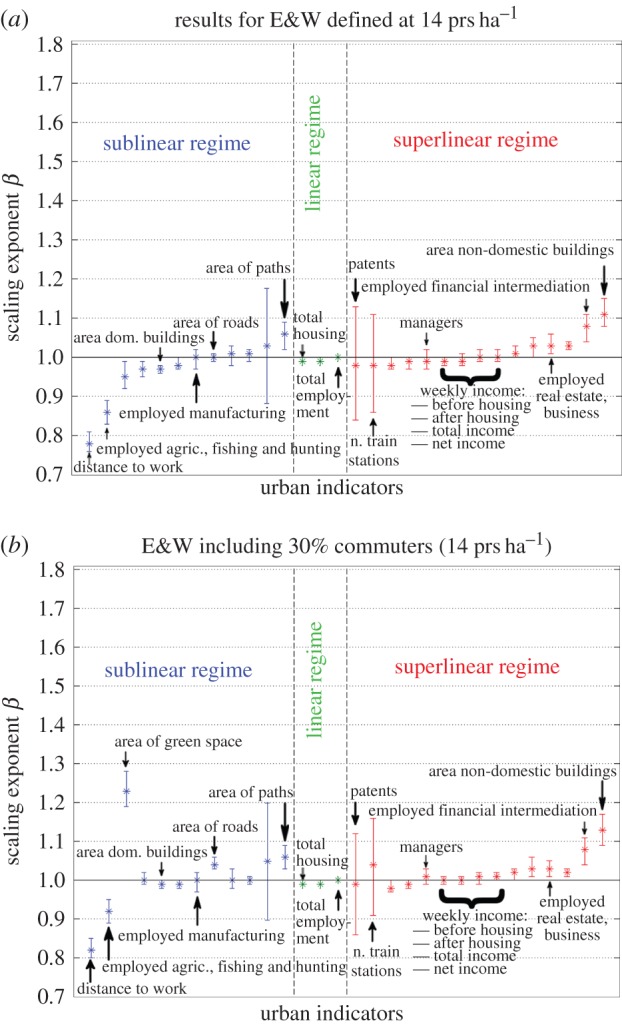
Scaling exponents with 95% CI for different urban indicators for cities defined at cut-off of *ρ*_c_ = 14 prs ha^−1^ in E&W without commuters (*a*) and with 30% commuters (*b*). (Online version in colour.)

The results of the analysis are summarized in figure 5*a* for cities, and in figure 5*b* for metropolitan areas. The details of the variables plotted in the figures are provided in electronic supplementary material, table S1.

We observe that for most measures, any deviations of the exponent *β* from linearity are extremely mild, and sometimes into the wrong regime, not corroborating the expected scaling laws. A clear illustration of this problem is given, for the sublinear regime, by the area of roads and by the area of paths; and for the superlinear regime, by the number of patents and by some employment categories, such as that for Managers. In §6, we will look in detail at patents, because these provide a clear example of the main issues that arise when trying to derive generic rules for urban indicators.

## 6. Patents

The number of patents produced is generally considered a proxy of the city's level of innovation. Nevertheless, there are many cities that do not have a single patent recorded over 10 years. Some of these cities have more than 1.8 × 10^5^ people, whereas many other small ones of less than 2 × 10^3^ inhabitants have patents registered.

In order to investigate the resilience and urban significance of the scaling exponent for this variable, we consider two scenarios. The first scenario corresponds to the urban system containing only cities larger than 10^4^ people, and the second scenario considers only cities larger than 5 × 10^4^ individuals. These two different population cut-offs are applied to the cities, and metropolitan areas defined above. In the literature, it is often the case that either of these two population cut-offs are employed to distinguish between a small settlement and a truly urbanized space.

**Figure 6.**
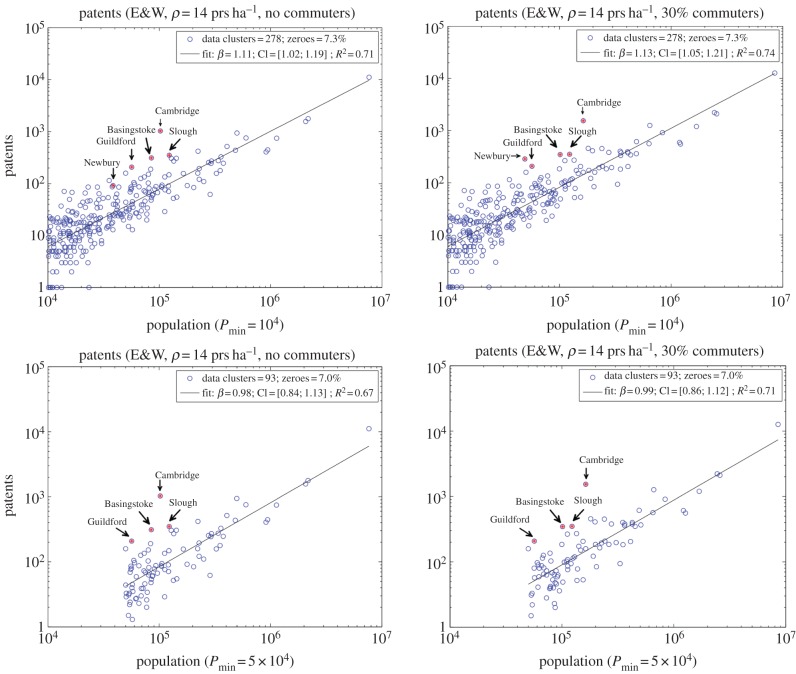
Scatter plots of patents for two different population size cut-offs. The top plots have a minimum population size of 10^4^ individuals, whereas the bottom ones have a cut-off of 5 × 10^4^ individuals. (Online version in colour.)

Scatter plots of patents and city size are shown in figure 6 for the two different cases. The plots show strikingly different results for the two population cut-offs. For the cut-off of 10^4^ individuals, the exponent lies within the superlinear regime (at a confidence level of 95%), whereas for the cut-off of 5 × 10^4^ individuals, linearity cannot be rejected. The absence of robustness for the scaling exponent for these two cases suggests that there is a lack of self-similarity for the full range of scales examined. This brings into question whether a minimum population size for settlements should or not be considered. Such a behaviour is often observed in systems that present power laws only for the tail of their distribution. Nevertheless, in this case, this variable has zero values for many of the clusters, leading to a substantial amount of zero counts, including cities as large as of the order of 10^5^ individuals. These are given in the form of percentages in the plot.

The sensitivity of the scaling exponent to the population size cut-off indicates, on the one hand, that the value of the exponent can bear no real significance on the behaviour of the system. On the other hand, this urban indicator is unable to present a quantifiable measure over 10 years for some large cities. This suggests that such a measure might be inadequate to properly quantify innovation.

In addition, the plots indicate that the most productive cities relative to size are not the biggest ones, but places that are highly rooted in education, such as Cambridge and Guildford or places corresponding to technological and business hubs. The latter are strategically located in the M4 corridor: e.g. Newbury (headquarters of Vodafone) and Slough (the largest industrial and business estate and headquarters of Telefonica 02), or are equally well connected to other strategic transport links within the Greater South East [29], such as Guildford (in addition to the university, it is also the headquarters of Philips) next to the M25 and Basingstoke (headquarters of many telecommunication companies) next to the M3. In this case, it is clear that in order to assess performance, one needs to go beyond size and consider path-dependencies.

## 7. Sensitivity analysis

In this section, we look at the sensitivity analysis of the scaling exponent *β*, to the different boundaries of cities and metropolitan areas. We make use of heat maps to illustrate the value of *β*, where the horizontal axis represents the parameter for the density threshold, and the vertical axis the parameter for the percentage of commuters in the clustering algorithms.

**Figure 7.**
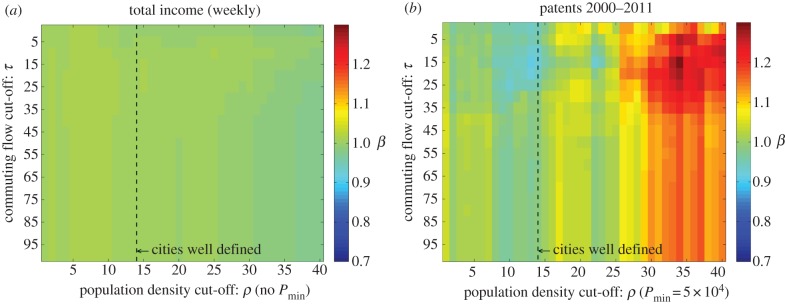
(*a*) Heat map for total income (no minimum population size imposed); (*b*) heat map for total number of patents for cities bigger than 5 × 10^4^ people.

The heat map in figure 7*a* clearly shows that for total income, population size does not convey any information on agglomeration effects, showing homogeneity throughout the map for all the different city demarcations. The same results were found for many other variables where superlinear exponents were expected, such as employment categories reflecting economic activity or requiring highly skilled individuals (see the electronic supplementary material, for more examples). On the other hand, the heat map in figure 7*b* shows that for variables that present nonlinear dependencies, such as patents, the scaling exponent is sensitive to boundary definitions.

## 8. Discussion

We showed that for all the different definitions of cities and metropolitan areas devised with our methodology, population size does not fully grasp the economic intricacies that constitute a system of cities in E&W. Looking at possible causes of discrepancy between our results and those previously found, it is evident that London plays a special role within the urban system of the UK. Its strong role as an information and economic hub suggests that the urban system is highly integrated and that it is difficult to partition the system into individual cities that capture these social interaction effects. On the other hand, if these economic functional areas are integrated following our methodology, we observe that for most urban indicators, London overperforms with respect to all other places in E&W. Its position as a primate city [30], but most importantly, as a world city in a relatively small country, could be affecting the entire urban system. The performance of cities such as London should possibly be evaluated relative to other global hubs operating within a larger-scaled network of interactions. Following Sornette's idea on the emergence of ‘big things' [31–33], a different perspective on the description of cities could be adopted, in which these global hubs are evaluated separately to their domestic counterparts. Sornette refers to the former as *dragon-kings*. A statistical test showing that London can be classified as such can be found in the electronic supplementary material. A two-system theory of cities might then emerge: a regime for cities driving international dynamics, the dragon-kings, and a regime for the remaining cities composing a country.

In addition to economic hubs, one also encounters knowledge hubs, which also present dragon-king-like qualities and which are not necessarily correlated with size. These hubs are the outcome of path-dependencies that give rise to emergent properties that are not present in all cities as is the case of patents. This is most dramatically demonstrated by the dominance of patent production in Cambridge, UK.

There are many difficulties in measuring the performance of a city through scaling laws. As discussed, there are problems in defining innovation in terms of patent counts, and this is not a unique case, other variables, such as CO_2_ emissions, present conflicting results. Some studies have found a sublinear relationship, whereas others have found a superlinear relationship between CO_2_ emissions and city size [34–37]. Such differences might stem from the nature of the measurement itself, whether the study refers to total or only transport emissions, and/or from qualitative differences between systems such as a country's level of development.

All this indicates that a theory of cities cannot rest simply on a relationship like equation (1.1), because relevant patterns pertaining to social behaviour, such as the well-known Pareto distribution of wealth, cannot be grasped if only aggregated values are considered. A theory of cities needs therefore to reproduce the main relevant emergent behaviours that are encoded in the diversity and heterogeneities of cities. It is only through this perspective that city planning and policy making can be effective.

## Endnotes

1 We exclude from the analysis the other two countries of the UK in order to avoid inconsistencies between the different datasets. The Office for National Statistics collects census data for England and Wales only, whereas two other different agencies collect data for Scotland and Northern Ireland separately.

2 The exponent was computed using the method for fitting a power-law distribution proposed in reference [26].

3 *N*_0_ = 0 individuals corresponds to the case where no cut-off is imposed, and all the settlements are taken into account.

4 If two or more clusters have the same largest flow, the ward is assigned to one of them at random.
